# Anaphylaxis in infants with cow's milk protein allergy: clinical aspects

**DOI:** 10.1186/2045-7022-3-S3-P119

**Published:** 2013-07-25

**Authors:** APBM Castro, DK Dias, LB Ferreira, GH Yonamine, CL Beck, AF Gushken, AC Pastorino, CMA Jacob

**Affiliations:** 1 Department of Pediatrics, Child’s Institute, São Paulo, Brazil; 2 Divisão de Nutrição, Child’s Institute, São Paulo, Brazil

## Background

Anaphylaxis is a severe manifestation of IgE mediated food allergy. There are few reports considering anaphylaxis in infants, a group where diagnosis and adequate management can be difficult. The aim of the study was to describe the clinical and laboratorial features of infants with cow's milk allergy (CMA) who had anaphylaxis.

## Methods

It was a retrospective study, including CMA patients who had anaphylaxis episodes before 2 years of age. Definition of anaphylaxis was according to the criteria proposed by Sampson et al, 2006. ImmunoCAP(ThermoFisher) was used for specific IgE analysis, and patients with values > 0.35kU/L were considered sensitized.

## Results

It was included 51 infants (31 M) with median age of first anaphylaxis episode at 6 mo (range 0.03-20 mo). Criterion 1 was the most prevalent (36 patients) at anaphylaxis diagnosis followed by criterion 2 (15 patients). Fresh milk was the trigger in 39 patients and dairy products in 12. Anaphylaxis was the first manifestation of CMA in 36 patients. There was recurrence of episodes in 37 patients, and in 46% it was related to intentional exposure. Many parents were unaware of the initial treatment of anaphylaxis (n = 19), 5 reported spontaneous resolution, but few parents reported use of parenteral medication (n=19) and only 5 informed that epinephrine was administered. Specific IgE to cow's milk was positive in 45/48 with a median of 13.4 kU/L (0.35 to 100 kU/L). Among three patients with ImmunoCAP to milk below 0.35, two had positive results in the second blood sample and the other had positive prick test. Positivity for casein occurred in 43 patients ranging from 0.35 to 100 (median 12.5 KU/L).

## Conclusion

Anaphylaxis can be a precocious manifestation of CMA and early recognition of this severe event was still undervalued by professionals and family members, with high recurrence rates. There was a reduced use of epinephrine, possibly as a consequence of lack of anaphylaxis identification or unknown of epinephrine as a first choice drug in anaphylaxis. Education strategies should be established for parents and physicians to minimize these events.

## Disclosure of interest

None declared.

